# Natural and Artificial Ventilation of Hospitals

**Published:** 1887-05-07

**Authors:** C. Theodore Williams


### NATURAL AND ARTIFICIAL VENTILATION OF HOSPITALS.

By C. Theodore Williams, M.A., M.D., F.R.C.P.

Mr. Keith D. Young's criticisms on my paper on the "Ventilation of Hospitals" merit some notice, both on account of his large experience in hospital construction, as an architect, and because of the important question they raise of Natural *v.* Artificial Ventilation. This was fully discussed at the meeting, and I hope my reply, as it appeared in your very able report, covered most of the objections raised to artificial ventilation. Mr. Young, when he states, "that the only hospital in London, besides Brompton, where artificial ventilation is in use is Guy's," ignores the Grocers' Wing of the London Hospital, where the same method of extraction is in use as at Brompton, and, I understand from some of the staff, works well. He also omits all mention of St. Thomas's Hospital, where artificial ventilation is certainly employed for extraction purposes, though possibly not for the supply of fresh air, each pavilion having an extraction-shaft through which flues from the basement furnaces pass, and thus form the source of extraction by heat. At Guy's there is no mechanical agency at work in the ventilation, which now seems to work to the satisfaction of the medical staff. Mr. Young asks, "Does not the experience of the large London hospitals show that it is possible to provide for an ample change of air in the wards by means of open windows, air-gratings at floor-levels, and smoke-flues?" and he himself answers the question most conclusively in the negative, by emphasising with me the necessity for maintaining at all times the temperature in the extraction-shaft. Now, how can this be done in summer, with no fires, and consequently no extracting current in the smoke-flues? for, after all, a bright burning fire with a narrow chimney-flue is as good an example of artificial ventilation as the most elaborate arrangement of shaft, and the position of the heating apparatus, whether at the top or at the bottom of that shaft, is not one of principle, but of detail.

To secure sufficient extraction all the year round, artificial ventilation becomes necessary, and it would be far better for some of the general hospitals if this were carried out. In many of them fires are lighted in the wards, even in summer, for invalid cooking and other purposes, and act as extractors. With regard to natural ventilation by opening windows, however well it may suit surgical cases, it does not answer always with medical ones; and many attacks of bronchitis and pulmonary congestion can be traced to this cause, of which Sir Andrew Clark gave a very startling example from his own wards, during the discussion.

